# Oncocytic variant, a novel subtype of chromophobe renal cell carcinoma: a report of two cases and a literature review

**DOI:** 10.1007/s13691-020-00459-7

**Published:** 2020-12-01

**Authors:** Takashi Karashima, Naoto Kuroda, Takahiro Taguchi, Hideo Fukuhara, Takahira Kuno, Kenji Tamura, Makoto Hiroi, Keiji Inoue, Tadanori Yamaguchi

**Affiliations:** 1 Department of Urology, Kochi Medical School, Kochi University, Kohasu, Oko, Nankoku, Kochi, 783-8505 Japan; 2 Department of Diagnostic Pathology, Kochi Red Cross Hospital, Kochi, 780-0062 Japan; 3 Human Health and Medical Science, Faculty of Medicine, Kochi University, Nankoku, 783-8505 Japan; 4 Laboratory of Diagnostic Pathology, Kochi Medical School Hospital, Nankoku, 783-8505 Japan; 5 Department of Cytopathology, Ayabe City Hospital, Ayabe, 623-0011 Japan

**Keywords:** Chromophobe renal cell carcinoma, Oncocytoma, Oncocytic variant

## Abstract

A novel variant of chromophobe renal cell carcinoma showing an oncocytic phenotype is proposed. Two new cases of this rare entity are presented and discussed along with six previous cases from our colleagues. A 76-year-old man and a 78-year-old man had a 3.4-cm and a 3.2-cm-diameter renal mass, respectively. On histopathological examination of surgical specimens, uniform eosinophilic cuboidal cells without a perinuclear halo growing in a tubular pattern were seen, and differential diagnosis from oncocytoma was necessary. Immunohistochemical staining for cytokeratin 7 and E-cadherin showed diffusely positive patterns in both, as in the previous reports. Although monosomy of chromosomes 7, 10, 13, and 17 was commonly observed in the previous reports, gains of chromosome 19 were observed in the two present cases. Immunohistochemical and cytogenetic approaches lead to exclusion of oncocytoma and the diagnosis of an oncocytic variant of chromophobe renal cell carcinoma.

## Introduction

Chromophobe renal cell carcinoma (ChRCC) is a rare variety of kidney neoplasm that accounts for approximately 5% of renal cell carcinomas (RCCs). ChRCC was first described in 1985 by Thoenes et al., who reported 12 cases of renal tumors consisting of chromophobe cells showing slightly opaque or finely reticular cytoplasm on hematoxylin and eosin staining [1]. Most ChRCCs are diagnosed at an earlier stage and show a better prognosis than conventional clear cell RCC. ChRCC is classified into three variants. The classic type, with more than 80% pale cells, is associated with necrosis and sarcomatoid changes with high growth and metastases. The eosinophilic variant, with more than 80% eosinophilic cells, shares certain characteristics with oncocytomas and shows nested, alveolar, or sheet-like architecture with eosinophilic granularity, perinuclear clearing, and peripheral accentuation of cytoplasm. The third variant is mixed [2]. Genetic abnormalities of ChRCC have been well described, with 70–90% of cases showing loss of chromosome 1, 2, 6, 10, 13, 17, or 21 [3, 4]. These genetic abnormalities might result in inactivated tumor suppressor genes, thus promoting tumorigenesis [5].

Renal oncocytoma is a benign neoplasm that consists of a pure population of oncocytes, which are well-differentiated, large neoplastic cells with an intensely eosinophilic granular cytoplasm as a result of a large number of mitochondria [6]. The origins of oncocytoma and ChRCC are the same, a collecting tubule [7], and their differential diagnosis depends on clinicopathological analysis.

Recently, a novel variation of ChRCC that morphologically resembles oncocytoma was reported [8, 9]. This rare variety exhibits oncocytoma-like histologic features, such as oncocytic cytoplasm, round central nuclei, absent perinuclear halo, and indistinct cell borders.

In this report, a total of eight cases, including two recent cases, of ChRCC showing oncocytic histological features are summarized, and a strategy for differential diagnosis of the oncocytic variant of ChRCC and oncocytoma using immunohistochemical and cytogenetic approaches is discussed. The oncocytic variant is proposed as the fourth variant of ChRCC, following the classic, eosinophilic, and mixed variants [8–10].

## Case presentation

**Fig. 1:**
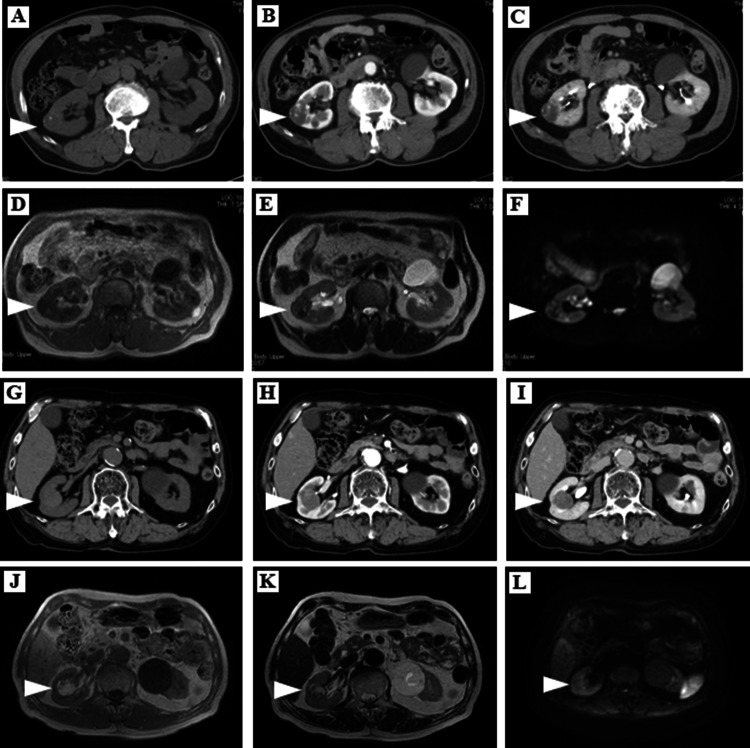
Preoperative diagnostic imaging of the first (**a**–**f**) and second (**g**–**l**) cases. In the first case, abdominal computed tomography (CT) shows a well-marginated exophytic right renal mass of 3.4 cm in diameter showing small calcifications in the plain phase (**a**), and a few are partly enhanced on both early- (**b**) and late-phase (**c**) contrast-enhanced CT. Magnetic resonance imaging (MRI) shows a regular isointense solid mass with partially high intensity on T1-weighted imaging (**d**) and irregularly high and low intensity on T2-weighted imaging (**e**) in the right kidney. Part of the mass shows high signal intensity on diffusion-weighted imaging (**f**). In the second case, abdominal CT shows an endophytic right renal mass, 3.2 cm in diameter, showing well-defined margins and faint contrast in the early (**h**) and late phases (**i**). Abdominal MRI demonstrates a high-intensity mass on T1-weighted imaging (**j**) and a low-intensity mass on T2-weighted imaging (**k**), and irregular signals with low and high intensity on T1- and T2-weighted imaging, respectively. No signal is identified on diffusion-weighted imaging (**l**)

A 76-year-old man and a 78-year-old man were referred to Kochi Medical School Hospital from private hospitals with incidental renal tumors, with diameters of 3.4 cm and 3.2 cm, respectively. The imaging findings of the two cases are presented in Fig. 1. Abdominal contrast-enhanced computed tomography showed well-defined tumor margins and no findings of metastases in both cases. Abdominal magnetic resonance imaging demonstrated a regular or irregular, low–high intensity on T1- and T2-weighted imaging. Part of the mass showed a high-intensity signal on diffusion-weighted imaging, suggesting a malignant neoplasm in the first case (Fig. 1f). No fatty component was observed on fat-suppression imaging.

**Fig. 2:**
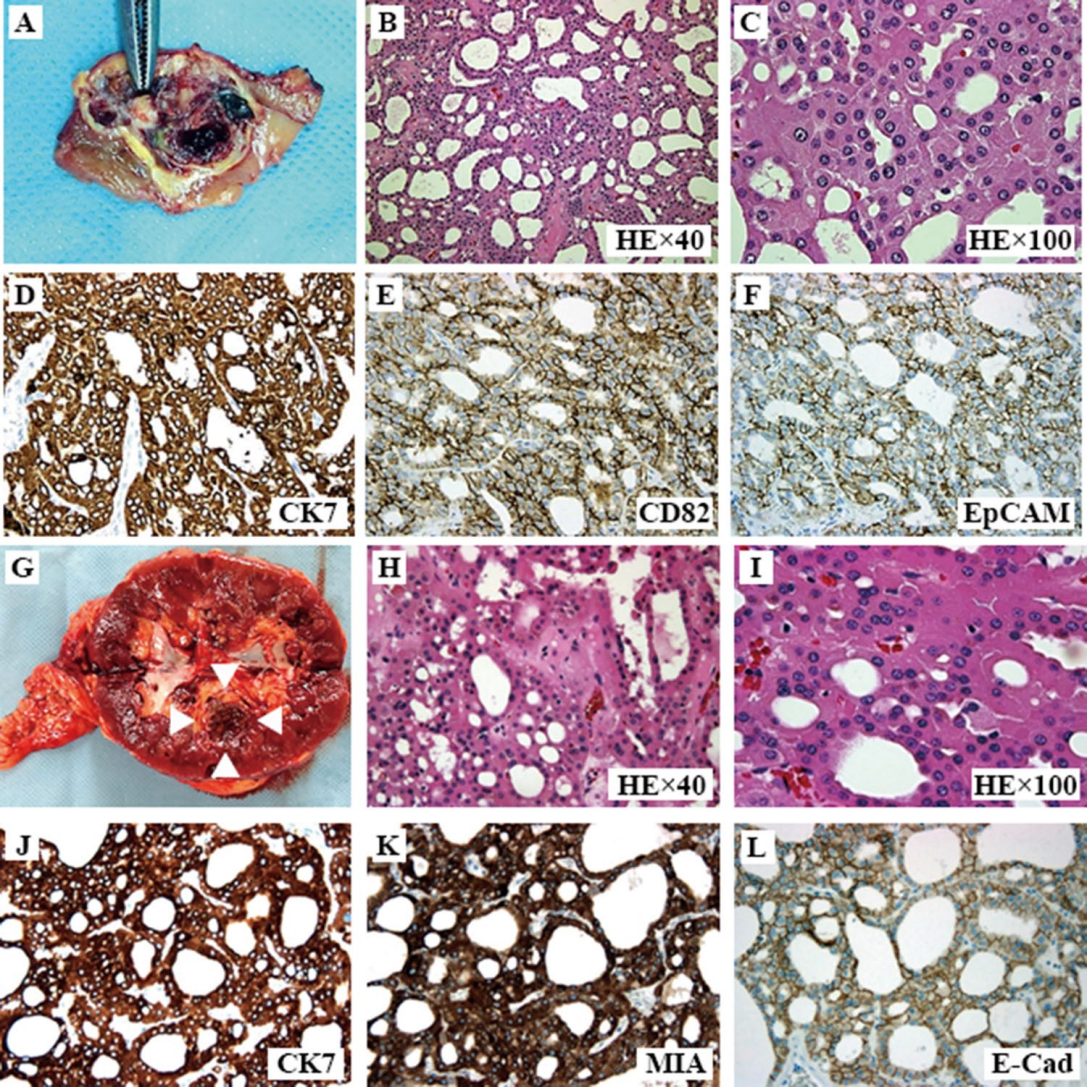
Macroscopic and microscopic findings of the tumors in the two present cases. Laparoscopic partial nephrectomy and laparoscopic radical nephrectomy were performed for the first and second cases, respectively. In the first case, the tumor is macroscopically well-marginated with a fibrous capsule. Necrosis and bleeding are identified. Calcifications are indicated with forceps (**a**). Microscopic findings with hematoxylin–eosin (HE) staining show uniform eosinophilic cuboidal cells with papillary and tubular growth (× 40) (**b**). Immobility of cell size and pale cells are not observed. Nuclei are centrally located and round, without a perinuclear halo. Few mitoses are identified (× 200) (**c**). Diffusely positive immunostaining for cytokeratin 7 (CK7) (**d**), cluster of differentiation 82 (CD82) (**e**), and epithelial cell adhesion molecule is observed (EpCAM) (**f**). In the second case, the tumor is a macroscopically well-circumscribed mass. The cross-sectional surface is mahogany brown, with bleeding in the center of the tumor (**g**). Microscopic findings with HE staining show uniform eosinophilic cuboidal cells with papillary and tubular growth (× 40) (**h**). Immobility of cell size or pale cells are not observed. Nuclei are centrally located and round without a perinuclear halo. Few mitoses are identified (× 200) (**i**). Diffusely positive immunostaining for CK7 (**j**) and mitochondria (MIA) (**k**) in the cytoplasm and E-cadherin (E-Cad) (**l**) in the cell membrane are observed

In the first case, the tumor was a macroscopically well-circumscribed, solid mass with a fibrous capsule. The cross-sectional surface was heterogeneously yellow–brown, lobulated, and separated with a septum. Bleeding and necrosis were identified (Fig. 2a–c). In the second case, the cross-sectional surface was mahogany brown, similar to oncocytoma, with bleeding in the center of the tumor. The pathological growth showed a commonly growing tubular pattern that was partly solid or cribriform. The tumor demonstrated uniform and hypereosinophilic cuboidal cells. Cell nuclei were round and centrally located, and no shrunken nuclei were observed. The cell borders were indistinct or slightly distinct (Fig. 2g–i).

Immunohistochemical staining showed diffusely positive patterns of cytokeratin 7 (CK7), cluster of differentiation 82 (CD82), epithelial cell adhesion molecule (EpCAM; MOC31), mitochondrial antigen (MIA), and E-cadherin (E-Cad) staining. The typical positive staining patterns for CK7, CD82, MIA, E-Cad, and EpCAM are presented in Fig. 2d–f and j–l.

**Fig. 3:**
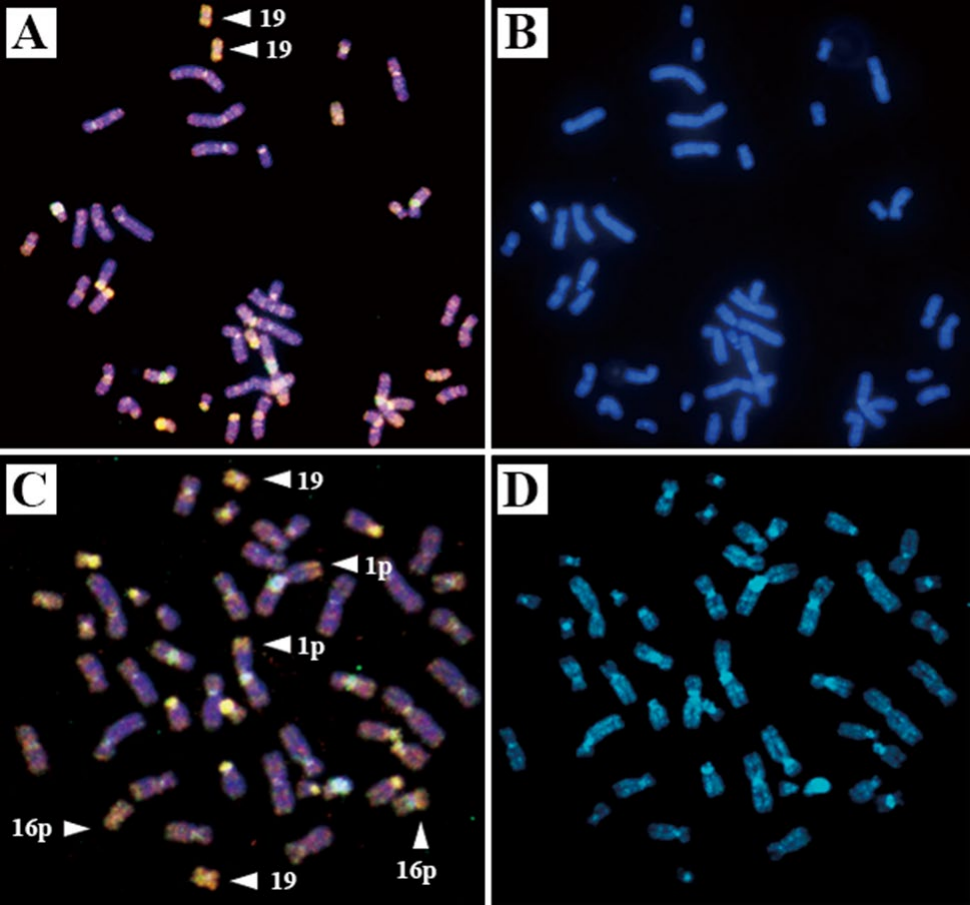
Cytogenetic analysis of the two present cases. A representative comparative genomic hybridization image of the tumor in the first (upper panels) and second (lower panels) cases. White arrows indicate amplified locations (orange signals; gain). Gains of chromosome 19 in the first case and gains of chromosomes 1p, 16p, and 19 in the second case are detected (**a**, **c**). Counterstaining with 4′,6-diamidino-2-phenylindole (blue signals) was performed for chromosome identification (**b**, **d**)

Chromosomal abnormalities, such as loss or gain, were detected by cytogenetic approaches. Comparative genomic hybridization (CGH) analysis demonstrated gains of chromosomes 1p, 16p, and 19, and no chromosome loss. Gain of chromosome 19 was a common abnormality in the two present cases (Fig. 3).

## Discussion

In 2010, a 76-year-old woman presented with a renal tumor showing morphological features indicative of oncocytoma, although the tumor was immunohistochemically positive for vimentin and CK7 and cytogenetically showed monosomy of chromosomes 7, 10, 13, and 17 by fluorescence in situ hybridization (FISH) analysis, corresponding to ChRCC [8]. An ‘oncocytic variant’ was then proposed as a novel subtype of ChRCC. Five more cases of the oncocytic variant of ChRCC were later reported in 2013 [9]. A similar variant of ChRCC was examined by cytological and ultrastructural approaches in 2015 [10]. The cases of the oncocytic variant of ChRCC are summarized in Table 1. Eight cases, including the two present cases, in Ayabe City Hospital, Kochi Red Cross Hospital, and our institution, are presented. The patients’ median age was 76 years (range: 64–82 years), and the male-to-female ratio was 5:3. Median tumor diameter was 3.95 cm (range: 2.0–11.0 cm). Four cases had undergone partial nephrectomy, and four cases had undergone radical nephrectomy. All cases were alive without recurrence after surgery at a median follow-up period of 24 months (range: 4–65 months). Tumor growth pattern, nuclear form, presence of cell border and vascular invasion, and immunohistochemical and cytogenetic findings are described below.

**Table 1:** Summary of eight cases of chromophobe renal cell carcinoma with oncocytic variant

No.	Age (years)	Sex	Tumor size (cm)	Therapy	Growth pattern	Nuclei			Cell border	VI	Immunohistochemistry					Cytogenetic findings	NED (months)
						Shrunken	Round	Location			CK7	CD82	MIA	E-Cad	CD10		
1^8^	76	F	7.0	Radical Nx	Tubular > solid	−	+	Central	ID–SD	+	d+	d+	d+	d+	−	Monosomy of 7, 10, 13, and 17^a^	65
2^9^	71	F	5.5	Radical Nx	Pure tubular	−	+	Central	ID–SD	−	d+	–	d+	d+	−	Monosomy of 7, 10, 13, 17, and 21^a^	26
3^9^	76	M	2.0	Partial Nx	Pure tubular	−	+	Central	ID–SD	−	d+	d+	d+	d+	−	Monosomy of 7, 10, 13, 17, and 21^a^	5
4^9^	64	M	4.5	Partial Nx	Tubular >> solid	−	+	Central	ID–SD	−	d+	f+	d+	d+	−	Monosomy of 7, 10, 13, 17, and 21^a^	26
5^9^	82	F	11.0	Radical Nx	Tubular >> solid	−	+	Central	ID–SD	−	d+	d+	d+	d+	−	Monosomy of 7, 10, 13, 17, and 21^a^	n.d.
6^10^	60	M	3.0	Radical Nx	Tubular or cribriform	−	+	Central	ID–SD	+	d+	d+	d+	d+	−	Monosomy of 7, 10, 13, and 17^a^	22
7*	76	M	3.4	Partial Nx	Tubular >> solid	−	+	Central	ID–SD	−	d+	d+	n.e.	d+	−	Gain of 19^b^	24
8*	78	M	3.2	Radical Nx	Tubular >> solid	−	+	Central	ID–SD	−	d+	n.e.	d+	d+	f.w.	Gain of 1p, 16p, and 19^b^	4
Median	76 (64–82)	M:F = 5: 3	3.95 (2.0–11.0)														24 (4–65)

*Nx* nephrectomy, + positive, − negative, *ID–SD* indistinct–slightly distinct, *M* male, *F* female, *VI* vascular invasion, *d* + diffuse positive, *f* + focal positive, *f.w.* focally weak, *n.e.* not examined, *NED* no evidence of disease, *n.d.* not described

*Present case

^a^Examined by fluorescence in situ hybridization

^b^Examined by comparative genomic hybridization

The histopathology of the tumor showed uniform and hypereosinophilic cuboidal cells commonly growing tubally with/without a solid or cribriform growth pattern. The cell border was slightly distinct or indistinct. Immobility of cell size and pale cells were not observed. Nuclei were centrally located and round without a perinuclear halo. No shrunken nuclei were observed. These phenotypes are common to oncocytoma, but not ChRCC. Colloidal iron stain showed focal to diffuse staining along the apical lumen (data not shown). Vascular invasion was identified in cases 1 and 6, which may suggest a malignant phenotype.

Immunohistochemical staining showed diffusely or focally positive patterns of CK7, CD82, MIA, and E-cadherin staining. These findings are common histological markers for diagnosing ChRCC. Negative to focally weak staining of the anti-cluster of differentiation 10 (CD10) antibody is another common finding. The negative staining of CD10 might contribute to the differential diagnosis of oncocytoma [11]. Negative staining of carbonic anhydrase 9 and RCC markers may rule out tumors derived from the renal proximal tubule, such as clear cell RCC and papillary RCC [12]. Negative anti-melanosome, cathepsin K, and transcription factor E3 (TFE3) staining may rule out Xp11.2 translocation RCC [13], and negative staining for alpha-smooth muscle actin may rule out renal angiomyolipoma [14].

Previous studies suggested the following features of this novel variant of ChRCC: (i) the tumor is predominantly composed of a tubular growth pattern, and a solid sheet pattern may be observed; (ii) the tumor cells are characterized by oncocytic cytoplasm, centrally located and round nuclei, an indistinct to slightly distinct cell border, and the absence of a perinuclear halo; (iii) the tumor cells are mostly diffusely positive for cytokeratin 7 and mitochondrial immunohistochemical staining; and (iv) chromosomal analyses show abnormalities. The recent two cases met all four of these criteria and are summarized, along with the other reported cases of ChRCC with oncocytic variant [8–10], in Table 1.

A cytogenetic and molecular approach can distinguish the variant of ChRCC. Although the previous six cases suggested that the presence of monosomy of chromosomes 7, 10, 13, 17, and/or 21 is promising for the diagnosis of ChRCC [3, 4], the recent two cases did not show chromosome loss and, instead, showed gains of chromosomes 1p, 16p, and/or 19. In the recent studies using CGH, it has been found that chromosomal gains can be detected more often in ChRCC than generally expected [15, 16]. Sperga et al. reported a high incidence of gains of chromosome 19 (59%) in ChRCC [17]. Renal oncocytoma generally shows a normal/diploid pattern and loss of chromosomes 1, 2, 8, 9, and 14, with a low incidence or random abnormality in a small number of chromosomes. No chromosome gains have been reported in oncocytoma to date [11, 18]. Thus, the present cytogenetic studies showing gains of chromosomes 1p, 16p, and 19 may lead to exclusion of oncocytoma. The present cytogenetic findings were based on CGH results, compared with previous studies that used FISH, and this difference in experimental approaches might be a contributing factor to these observations.

Using histology, this rare variant of ChRCC must be distinguished from renal oncocytoma, the eosinophilic variant of ChRCC, sporadic hybrid oncocytic/chromophobe tumor, the solid variant of oncocytic papillary RCC, oncocytoma-like renal angiomyolipoma, and oncocytoid RCC after neuroblastoma. The absence of a cell border, perinuclear halo, wrinkled/raisinoid nuclei, foamy macrophages in the stroma, or focal papillary growth pattern, and diffusely positive CK7 staining lead to a final diagnosis of the oncocytic variant of ChRCC [19, 20].

The categorization of the tumors as benign or malignant remains controversial. The renal tumor that Trpkov et al. designated ‘low-grade oncocytic tumor’ characterized by a CD117−/CK7+ immunoprofile may be the same as the present proposed variant of chromophobe RCC [21]. Clinically, these tumors may be categorized between benign oncocytoma and malignant chromophobe RCC. Additional investigations of more cases are needed.

Two new cases diagnosed as the chromophobe subtype were presented as malignant renal neoplasms showing benign neoplasia similar to oncocytoma. These two entities of tumors are often confused. In the past, some of the cases diagnosed as recurrent or metastatic oncocytoma might have been the oncocytic variant of ChRCC. Immunohistochemical and cytogenetic findings allow the differential diagnosis of kidney neoplasms with rich variations, such as ChRCC.

## Data Availability

Requests for the study materials and dataset used to support the conclusions of this article should be directed to the corresponding author.

## Abbreviations

CD10: Cluster of differentiation 10
CD82: Cluster of differentiation 82
CGH: Comparative genomic hybridization
ChRCC: Chromophobe renal cell carcinoma
CK7: Cytokeratin 7
E-Cad: E-cadherin
EpCAM: Epithelial cell adhesion molecule
FISH: Fluorescence in situ hybridization
MIA: Mitochondrial antigen
RCC: Renal cell carcinoma
TFE3: Transcription factor E3
